# The Central Portion of Factor H (Modules 10–15) Is Compact and Contains a Structurally Deviant CCP Module

**DOI:** 10.1016/j.jmb.2009.10.010

**Published:** 2010-01-08

**Authors:** Christoph Q. Schmidt, Andrew P. Herbert, Haydyn D.T. Mertens, Mara Guariento, Dinesh C. Soares, Dusan Uhrin, Arthur J. Rowe, Dmitri I. Svergun, Paul N. Barlow

**Affiliations:** 1 Edinburgh Biomolecular NMR Unit, Centre for Chemical and Translational Biology, Schools of Biological Sciences and Chemistry, University of Edinburgh, Edinburgh EH9 3JJ, UK; 2 European Molecular Biology Laboratory—Hamburg Outstation, c/o DESY, Notkestrasse 85, 22603 Hamburg, Germany; 3 Medical Genetics Section, Molecular Medicine Centre, Institute of Genetics and Molecular Medicine, Western General Hospital, University of Edinburgh, Crewe Road, Edinburgh EH4 2XU, UK; 4 NCMH Business Centre, School of Biosciences, University of Nottingham, Sutton Bonington, Leicestershire LE12 5RD, UK

**Keywords:** CCP, complement control protein, fH, complement factor H, fB, complement factor B, SAXS, small-angle X-ray scattering, AUC, analytical ultracentrifugation, HSQC, heteronuclear single quantum coherence, NOE, nuclear Overhauser effect, PBS, phosphate-buffered saline, PDB, Protein Data Bank, EOM, even-and-odd mode, TOCSY, total correlated spectroscopy, SA, solvent-accessible surface area, complement system, short consensus repeat, NMR structure, small-angle X-ray scattering, analytical ultracentrifugation

## Abstract

The first eight and the last two of 20 complement control protein (CCP) modules within complement factor H (fH) encompass binding sites for C3b and polyanionic carbohydrates. These binding sites cooperate self-surface selectively to prevent C3b amplification, thus minimising complement-mediated damage to host. Intervening fH CCPs, apparently devoid of such recognition sites, are proposed to play a structural role. One suggestion is that the generally small CCPs 10–15, connected by longer-than-average linkers, act as a flexible tether between the two functional ends of fH; another is that the long linkers induce a 180° bend in the middle of fH. To test these hypotheses, we determined the NMR-derived structure of fH12–13 consisting of module 12, shown here to have an archetypal CCP structure, and module 13, which is uniquely short and features a laterally protruding helix-like insertion that contributes to a prominent electropositive patch. The unusually long fH12–13 linker is not flexible. It packs between the two CCPs that are not folded back on each other but form a shallow vee shape; analytical ultracentrifugation and X-ray scattering supported this finding. These two techniques additionally indicate that flanking modules (within fH11–14 and fH10–15) are at least as rigid and tilted relative to neighbours as are CCPs 12 and 13 with respect to one another. Tilts between successive modules are not unidirectional; their principal axes trace a zigzag path. In one of two arrangements for CCPs 10–15 that fit well with scattering data, CCP 14 is folded back onto CCP 13. In conclusion, fH10–15 forms neither a flexible tether nor a smooth bend. Rather, it is compact and has embedded within it a CCP module (CCP 13) that appears to be highly specialised given both its deviant structure and its striking surface charge distribution. A passive, purely structural role for this central portion of fH is unlikely.

## Introduction

The complement system is a major component of mammalian immune defences.^1,2^ Complement activation not only leads to assembly of cytolytic membrane attack complexes^3^ but also augments acquired immunity and generates mediators of inflammation and opsonisation. The phylogenetically ancient complement system employs comparatively rudimentary mechanisms for discrimination between self and non-self.^4^ These are based largely upon the regulators of complement activation^5,6^ protein family, of which complement factor H (fH) is a prominent member.^7^ Mutations and polymorphisms in the fH gene are linked to kidney diseases (atypical haemolytic uraemic syndrome and dense deposit disease) and age-related macular degeneration (reviewed by de Cordoba and de Jorge^8^) and, tentatively, to heightened risks of Alzheimer's disease^9^ and myocardial infarction.^10,11^

Factor H is an abundant 155-kDa (1213-amino-acid residue) plasma glycoprotein.^12^ It competes with the pro-enzyme complement factor B (fB) for binding to molecules of the pivotal complement protein C3b. C3b, a proteolytic fragment of C3, is generated on a continuous low-level basis by the alternative pathway of complement. Nascent C3b readily binds covalently to any nearby surfaces, where it triggers several inflammatory events.^13^ Binding of fB to C3b and subsequent cleavage of fB yield C3b.Bb, which is a C3 convertase. This bimolecular enzyme cleaves C3 to form further molecules of C3b, thereby stoking a positive feedback loop that rapidly amplifies the number of C3b molecules.^14^ Binding of fH to C3b, on the other hand, mediates destruction of C3b by factor I. Factor H is also able to accelerate an irreversible disassembly of C3b.Bb, providing further regulatory activity. Through poorly understood mechanisms, fH engages more effectively with C3b (or C3b.Bb) that is bound to self-surfaces bearing polyanionic markers such as glycosaminoglycans and sialic acid, than with these same proteins when they are deposited on foreign surfaces.^15^ In this way, fH acts selectively to protect host tissue.

**Fig. 1:**
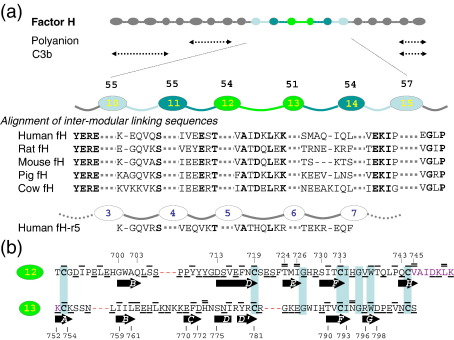
Summary of human fH and its orthologues. (a) CCPs are represented by ovals sized to reflect the number of residues that each contains (51–62 residues); intermodular linker lengths (from three to eight residues) and ligand-binding regions are also summarised. Modules 10–15 are highlighted; the blow-up displays the number of residues in each CCP and the aligned sequences of linkers within orthologues. Also shown is an equivalent region (CCPs 3–7 out of nine CCPs) in human fH-related protein 5 (labelled fH-r5). (b) Sequence of CCPs 12 and 13 aligned based on their structures.^19^ β-Strands are indicated by arrows and by the first and the last residue numbers. Shaded boxes highlight identities. Underscored residues are exposed, an overscore identifies a buried residue, and a double overscore indicates a residue buried at the intermodular interface.^20^

The intact fH protein molecule has not been crystallised and is too large for structure determination by nuclear magnetic resonance (NMR). It is unusually composed entirely of numerous^16,17^ examples of the complement control protein (CCP) module (∼ 60 residues, also known as a short consensus repeat or sushi domain);^18^ there are 20 CCPs in fH orthologues from mice to humans (Fig. 1). Three-dimensional structures of 12 of the fH CCPs (in fragments containing up to four CCPs), showing that they approximate to prolate spheroids of ∼ 3.8 nm by ∼ 1.6 nm, have been reported.^21^ Transmission electron microscopy,^22^ small-angle X-ray scattering (SAXS), and analytical ultracentrifugation (AUC)^23^ of fH are consistent with a molecule of about half the length that would be expected, were all CCPs stretched out in a fully extended head-to-tail arrangement. Thus, each module might be strongly tilted with respect to its neighbours, or the chain of CCPs might double back on itself at a bend imposed by the middle section of the molecule.

Factor H has two C3b-binding regions (Fig. 1), one towards either end of the molecule.^24–27^ The main binding sites for C3b lie within CCPs 1–4 (with CCPs 1–3 retaining residual binding affinity^28^) and CCPs 19–20. These two sites must cooperate, since the surface-plasmon-resonance-derived binding affinity of intact fH for immobilised C3b is 0.6–1.6 μM, which is significantly tighter than either site alone (4–10 μM).^27^ Other potential C3b-binding sites—encapsulated in fH6–8^25^ and fH8–15^29^—exhibited only weak or very weak affinities, respectively, according to a recent report.^27^ To help discriminate between C3b and C3b.Bb according to context (on self-surfaces *versus* non-self-surfaces), two additional binding sites on fH (one within CCPs 6–8^30,31^ and the other within CCPs 19–20^32,33^) recognise polyanions such as glycosaminoglycans and sialic acids.^34,35^ These ligand-binding regions correspond to locations of disease-linked single-nucleotide polymorphisms and mutations^8^—e.g., Y402H in module 7 is a major risk factor for age-related macular degeneration,^36^ whilst many mutations in CCPs 20 are linked to atypical haemolytic uraemic syndrome.^37^

An important question as to how the four principal ligand-binding sites encompassed in CCPs 1–8 and 19–20 work in a cooperative fashion remains.^21^ Intervening modules are likely to be critical in this respect. An often-noted feature of the fH central region is the preponderance of relatively small modules joined by comparatively long linking sequences (Fig. 1; for simplicity in this work, we consider module boundaries as the first and the last of the four conserved cysteines, unless otherwise stated). This, taken together with the absence of strong evidence for binding sites amongst these modules, has led to speculation that the central CCPs of fH act primarily as a flexible tether between N-terminal and C-terminal regions constraining, but not directing, the spatial distance between them; an orthogonal viewpoint is that the central CCPs cause the fH molecule to bend back on itself in a defined way, thus organising N and C termini into a multivalent composite recognition and binding site.^23,30,38,39^ In previous work, a version of fH from which modules 11–15 had been deleted proved not to be fully active but retained some limited functionalities.^25^ Its affinity for C3b was not quantified, however; thus, whether its N-terminal and C-terminal functional regions could cooperate is not known.

We set out to address the extent to which the coincidence of long linkers and small modules constitutes a functionally relevant architectural feature at the centre of the fH molecule. We therefore determined the solution structure and dynamics of the 12th and 13th CCPs of factor H (fH12–13), since this segment contains both the smallest module (CCP 13 contains just 51 residues) and the longest intermodular linker (eight residues) within the entire RCA family. This information was supplemented with low-resolution structural data for segments of fH extended by one or two modules on either side of fH12–13 (i.e., fH11–14 and fH10–15). We obtained evidence that, far from introducing flexibility, long linkers induce defined tilt angles between CCP modules.

## Results

### Solution NMR studies reveal a rigid fH12–13 structure

**Fig. 2:**
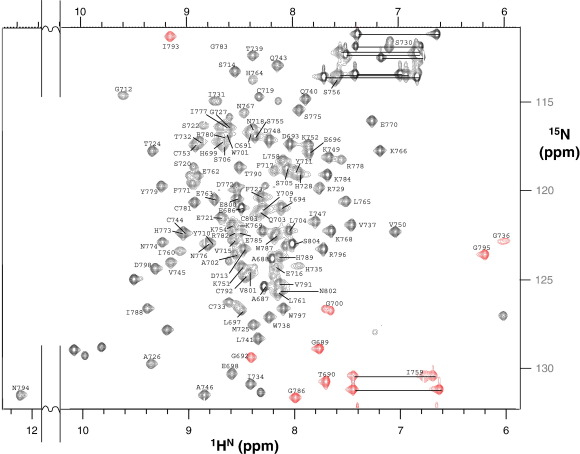
Assigned ^1^H,^15^N HSQC spectrum of fH12–13. See the text for sample conditions and data collection parameters. Alphanumeric characters indicate assignments. Spectrally folded peaks are shown in red. Paired asparagine and glutamine NH_2_ resonances are joined by dotted lines.

**Fig. 3:**
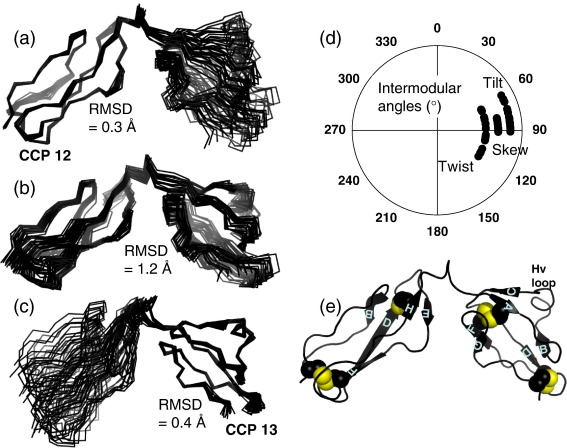
Ensemble of NMR-derived structures. Backbone overlays (and RMSDs) of the 20 lowest-energy structures selected from the 100 calculated. (a) Overlaid on module 12. (b) Overlaid on module 13. (c) Overlaid on both CCPs (RMSD per residue plotted in Fig. 4). (d) Summary of intermodular angles for ensemble. (e) Cartoon (PyMOL: http://www.pymol.org) of the nearest-to-mean structure; disulphides are highlighted by sphere representations of sulphur atoms. β-Strands (from STRIDE^41^) are labelled on the basis of alignment (data not shown) with other CCP structures and convention based on the occurrence of a maximum of eight strands (A–H) in any given CCP.^18^

Good-quality NMR spectra of ^15^N-labelled and ^15^N,^13^C-labelled fH12–13 [illustrated in the ^15^N,^1^H heteronuclear single quantum coherence (HSQC) spectrum in Fig. 2] allowed near-complete assignment of ^1^H, ^15^N, and ^13^C nuclei. The oxidised state of the eight Cys residues was confirmed by mass spectrometry, whilst the disulphide pattern (Cys^I^-Cys^III^ and Cys^II^-Cys^IV^) was inferred from the proximities of cysteine pairs in initial structures calculated without disulphides; these were then included^40^ in subsequent calculations. The final ensemble of the 20 lowest-energy structures, determined on the basis of 2911 nuclear Overhauser effect (NOE)-derived restraints, converges well (Table 1). Predictably, overlays of individual modules (Fig. 3a and b) yield better local convergence than overlays of both CCPs at once (Fig. 3c). Nonetheless, the 1.2-Å backbone root-mean-square deviation (RMSD) for the ensemble of bimodules is not consistent with a large variation in intermodular orientations (Fig. 3d); this tallies with the relatively plentiful NOEs detected within the linker, between the linker and modules, and between modules (Table 1). Strands and connecting loops in each of modules 12 and 13 are labelled (Fig. 3e), in accordance with a convention based on the occurrence of a maximum of eight β-strands (A–H) in some individual CCPs.

**Fig. 4:**
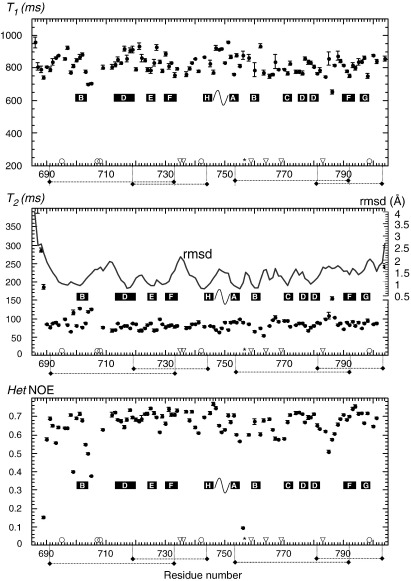
Relaxation data for fH12–13. *T*_1_, *T*_2_, and ^1^H,^15^N NOE values plotted against residue number. The central panel incorporates, in addition to *T*_2_ values (left-hand *y*-axis), the per-residue RMSD (right-hand *y*-axis) for the ensemble (overlaid on the bimodule). On each panel, β-strands (numbered according to the convention used in Fig. 3) are summarised; a curvy line represents the intermodular linker. Along the *x*-axes, circles indicate residues giving rise to broad or weak NH signals from which no measurements could be made; triangles correspond to prolines; and (^⁎^) labels the amide that was not found in the HSQC.

Relaxation measurements (Fig. 4) reveal mobility in the A–B loop of CCP 12 and its short “hypervariable region” beyond strand B (e.g., Leu704 and Ser705 have small ^15^N *T*_1_/*T*_2_ ratios and low ^1^H,^15^N NOEs). The very short A–B loop of CCP 13 also appears mobile (Ser756 has a small ^1^H–^15^N NOE, backbone NH signals of Asn757 could not be found, and only a weak HSQC peak was detected for Ile759 NH). The unusual helix-like insertion in the hypervariable region of CCP 13 (discussed below) is not particularly mobile on timescales probed by measurements of ^1^H,^15^N NOEs and *T*_1_/*T*_2_, but a stretch of residues just beyond the insertion and looping around to CCP 13 strand C exhibits below-average ^1^H,^15^N NOEs (Asn767, Lys768, and Glu770) and a very weak HSQC peak (Lys769). The D–E loop of CCP 13 appears flexible; Gly783 has a weak HSQC peak, Glu785 has exceptionally low ^1^H,^15^N NOE, and Gly786 NH has the lowest *T*_1_/*T*_2_ ratio of any residue apart from unstructured termini. Of particular interest is that residues of the linking sequence between modules appear immobile on the timescales probed; six out of eight linker residues have average or higher-than-average ^1^H,^15^N NOEs, whilst *T*_2_ values are just below average, and *T*_1_ values are typically just above average. This is consistent with the aforementioned numerous ^1^H–^1^H NOEs involving linker residues and the relatively low RMSD obtained upon overlaying of the bimodular ensemble (Fig. 3c); taken together, these data imply that CCPs 12 and 13 are quite rigidly associated, and that the ranges of tilt, twist, and skew intermodular angles in Fig. 3d (and Table 1) are not a consequence of overrefinement in the ensemble of final structures.

### The structures of modules 12 and 13

**Fig. 5:**
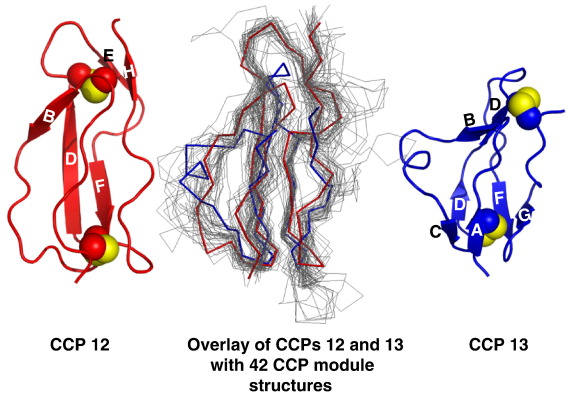
Comparisons of CCPs 12 and 13 with a set of known CCP structures. Cartoon representations (PyMOL) of CCPs 12 and 13 flank a C^α^ trace overlay (generated using the program MAMMOTH-mult^19^) of all CCPs with experimentally derived three-dimensional structures from the complement system. Highlighted within the overlay are CCP 12 (red) and CCP 13 (blue).

**Fig. 6:**
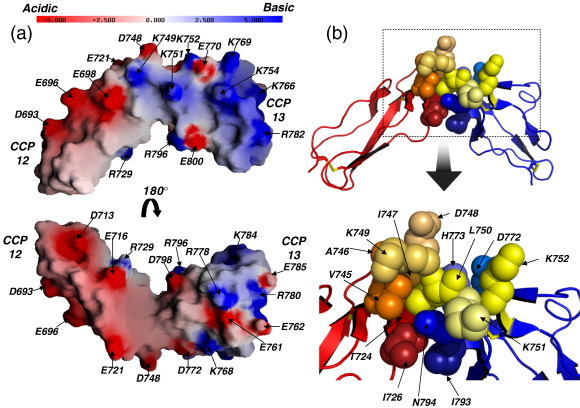
Illustrations of the electrostatic surface and intermodular interface of fH12–13. (a) Electrostatic surface^43^ (top; same view as in Fig. 3e); CCP 12 is predominantly electronegative, whilst the linker and CCP 13 display an electropositive patch that includes the helix-like hypervariable region. (b) In this cartoon (PyMOL), disulphides are drawn as sticks (yellow sulphur atoms), CCP 12 is shown in red, CCP 13 is shown in blue, and linker is shown in yellow/orange. Different shades of these colours are used to distinguish side chains (drawn as spheres; heavy atoms only) contributing to the intermodular interface. Side chains are labelled in the blow-up (bottom).

Structure determination of fH12–13 reveals an archetypal CCP in module 12 that overlays very well (C^α^RMSD < 2 Å) with 14 out of 42 experimentally determined CCP structures (Fig. 5); it superimposes particularly well (C^α^ RMSD = 1.4 Å) with CCP 19 of fH (both CCPs are in sequence cluster B in accordance with Soares *et al.*^42^). Notable features include a *cis*-Pro at 708, the unusual occurrence of a Trp in strand B, and three successive Tyr residues prior to strand D. Its surface is predominantly electronegative (Fig. 6), with dispersed patches of lipophilicity.

Module 13 is structurally divergent (Fig. 5) and overlays poorly with CCP 12 (C^α^ RMSD over a span of 46 aligned residues, 3.5 Å); at 51 residues, CCP 13 is the smallest CCP known, but it does not overlay well with another small (53-residue) CCP (CCP 4 of fH; C^α^ RMSD = 2.8 Å) or 36 other solved CCP structures (RMSD > 3.0 Å). The long axis of CCP 13 is about 4 Å shorter than that of CCP 12, as is the distance between disulphide bridges, whereas its diameter is about 2.5 Å greater; hence, it is more spherical than CCP 12 (Fig. 5). Surprisingly, an insertion of three residues (^766^KNK^768^) occurs within the hypervariable loop of CCP 13 that projects in a helical-like structure laterally from the module. Together with lysines in and just beyond the linker (and basic side chains from elsewhere), this bulge on the side of module 13 contributes to a strikingly electropositive patch extending over almost an entire face of the module (Fig. 6). A three-residue deletion occurs within the A–B loop of CCP 13, and a three-residue deletion occurs in its flexible D–E loop. Strand D is interrupted, whilst hydrophobic-residue-rich strand E of CCP 12 is replaced in CCP 13 by an exposed loop (^783^GKE^785^), and there is no β-strand H in CCP 13 (refer to Fig. 5). The D–E loop, together with the C-terminal (strand H) region of CCPs, is normally essential for forming and stabilising the interface with the following module. It is therefore clear that either the 13–14 interface must be flexible or modules 13 and 14 adopt an arrangement that is distinct from those of other module pairs. In summary, CCP 13 appears highly specialised with regard to its overall shape, electrostatic properties, and interface with the following module.

### Module 13 is tilted by ∼ 80° with respect to module 12

Four (Val745, Ala746, Ile747, and Leu750) of the eight linker residues bury all or a substantial portion of their hydrophobic side chains in the interface between modules along with two residues (Thr724 and Ile726) from CCP 12 strand E and residues from the C–D loop (His773) and the F–G loop (Asn794 and, to a lesser extent, Ile793) of CCP 13 (Figs. 1 and 6). The β-methylenes of the other linker residues (and in particular Lys752) also contribute to this miniature core, which forms a wedge between the modules. Altogether, ∼ 560 Å^2^ of surface area may be considered buried between modules. The hydroxyl group of Thr724 and the HN^ɛ^ of His773 are within H-bonding distance, as are (in the lowest-energy structure) the side-chain amide of Asn794 and the backbone nitrogen of the linker residue Lys751.

**Fig. 7:**
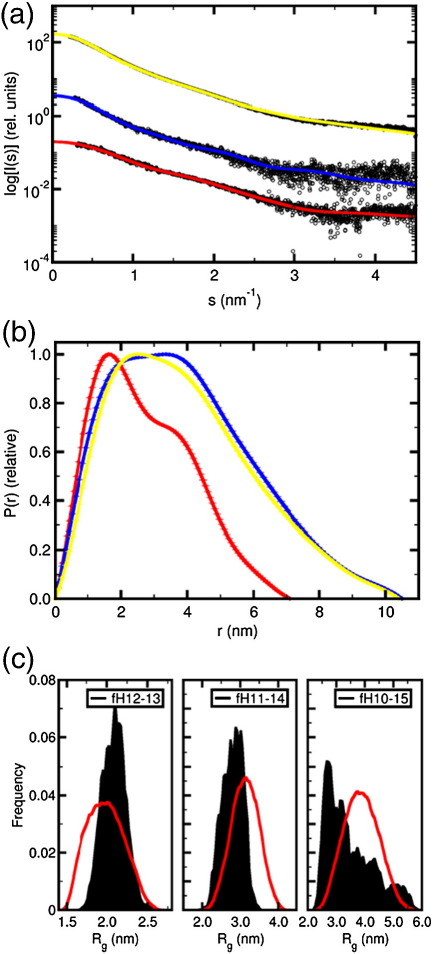
Overview of SAXS data and analysis. (a) Scattering curves for fH12–13 (red), fH11–14 (blue), and fH10–15 (yellow). Continuous lines represent fits obtained by CRYSOL for the best fH12–13 NMR model, or by rigid-body modelling (BUNCH) for fH11–14 and fH10–15; curves have been arbitrarily displaced along the logarithmic axis for clarity. (b) *p*(*r*) functions (arbitrary units) for fH12–13 (red), fH11–14 (blue), and fH10–15 (yellow), computed from X-ray scattering patterns using GNOM. (c) Radius-of-gyration distributions of pools (red lines) and selected structures (black) for fH12–13, fH11–14, and fH10–15 using EOM. Integral of area defined by histograms = 1.

The software CRYSOL was employed to fit each conformer from the NMR-derived ensemble of fH12–13 to SAXS data collected on fH12–13 (in 50 mM potassium phosphate, pH 7.4). Good fits (see Fig. 7a) of the scattering data to each ensemble member were observed (1.24 < χ < 1.43), supporting the structure determined by NMR (in 20 mM potassium phosphate, pH 6.6).

### Size-exclusion chromatography and AUC consistent with tilted modules in fH10–15

When passed down a calibrated size-exclusion chromatography column, fH12–13 eluted a little later than globular proteins of a comparable molecular mass, consistent with the slightly elongated protein molecule observed by NMR. On the other hand, the six-module construct fH10–15 also eluted at a position similar to that of a comparable globular protein; thus, the overall shape of fH10–15 is not highly elongated (data not shown).

In a more rigorous follow-up study, fH12–13, fH11–14, and fH10–15 were submitted to analysis via sedimentation–velocity AUC, and the results are summarised in Table 2. Analysis of AUC profiles demonstrated that all three of the samples are predominantly monomeric at 0.2–0.4 mg ml^− 1^ in phosphate-buffered saline (PBS). As may be seen from Table 2, the fitted frictional ratios indicate that fH11–14 is considerably more elongated in shape than fH12–13. On the other hand, a very interesting feature of the AUC results is that fH10–15, whilst more extended than fH12–13, is less elongated in overall shape than fH11–14. A speculative suggestion is that whilst all the modules are tilted with respect to their neighbours, they are not all tilted in the same direction. This suggestion could have been interrogated further by utilising the AUC data to construct bead-based models, but this would have entailed a more extensive study, requiring chemical constraints as opposed to purely geometrical constraints. It was therefore decided to subject the samples to SAXS on the basis that the latter technique provides a larger number of geometrical parameters upon which to base molecular modelling.

### SAXS confirms the compact structure of fH10–15

The experimental scattering patterns of fH12–13, fH11–14, and fH10–15 are presented in Fig. 7a, and parameters determined from these data sets are listed in Table 2. The values of molecular mass and hydrated particle volume estimated from the scattering data agree with those predicted from the sequences for all constructs. Thus, in agreement with the AUC data (collected on samples in PBS, pH 7.3), SAXS (collected on samples in 50 mM potassium phosphate, pH 7.4) showed that all constructs appear to be monomeric in solution. To further cross-validate the consistency of the structural and hydrodynamic data, we computed the sedimentation coefficients of the bead models generated by SAXS for the three fH constructs using the program HYDROPRO.^44^ These computed values (Table 2) are in very good agreement with the experimental values, indicating that the different experimental conditions and ionic strengths employed in the structural and hydrodynamic experiments do not lead to significant changes in the overall structure.

Analysis of the radii of gyration (*R*_g_) and maximum dimensions *D*_max_ of the fH constructs reveals that fH11–14 is significantly more extended than fH12–13, but that both fH11–14 and fH10–15 share a *D*_max_ of ∼ 10.5 nm. The data thus indicate that (i) modules 11–14 do not lie in a smooth curve reminiscent of a horseshoe (more likely, they adopt a zigzag arrangement); and (ii) the terminal CCP modules of the fH10–15 construct (i.e., CCPs 10 and 15) are tilted towards the more central modules, forming an overall compact structure.

The distance distributions *p*(*r*) for all fH constructs are positively skewed, with tails at large distances (Fig. 7b), indicating that all the particles have non-spherical elongated shapes. The fH12–13 data show a bimodal distribution characteristic of dumbbell-shaped particles, with the first maximum at ∼ 1.6 nm and a second maximum at ∼ 3.4 nm. Along with a maximum size *D*_max_ of 7.1 nm, these distances are in good agreement with the ensemble of NMR-derived structures; 1.6 nm corresponds approximately to the radii of the individual CCP 12 and CCP 13 modules, and 3.4 nm corresponds very approximately to the separation of the centres of mass of the CCPs.

Both the two larger constructs fH11–14 and fH10–15 are more extended in shape than fH12–13. The position of the second maximum in the distance distributions of all three constructs is conserved, indicating that the average intersubunit distance is maintained at ∼ 3.4 nm. However, the position of the first maximum is shifted to larger distances in fH11–14 and fH10–15, corresponding to an increase in the effective cross section of these constructs and the formation of compact structures.

### SAXS suggests that fH12–13, fH11–14, and fH10–15 lack intermodular flexibility

An analysis of intermodular dynamics in fH12–13, fH11–14, and fH10–15, based on the SAXS data, was conducted using the recently developed ensemble optimisation method.^45^ For each construct, the ensemble optimisation method analysis produced a skewed narrow *R*_g_ distribution for the selected ensemble of structures that best fit the SAXS data (Fig. 7c). These distributions may be compared with the broad range of *R*_g_ exhibited by the initial pool of fH12–13, fH11–14, and fH10–15 conformers (Fig. 7c) with randomly generated intermodular angles; such a comparison suggests that in none of these cases are modules free to articulate at random relative to their neighbours.^45,46^ The selected ensemble for fH12–13 is skewed toward higher values of *R*_g_ [*R*_g_(av) = 2.1±0.1 nm], confirming that this structure is significantly more extended than an average random configuration. Conversely, the selected ensembles for both fH11–14 and fH10–15 are skewed towards lower values of *R*_g_ [*R*_g_(av) = 2.8 ± 0.3 nm and *R*_g_(av) = 3.5 ± 0.8 nm, respectively], indicating that these structures are much more compact than those in the random pool.

### Modelling structures based on SAXS and NMR-derived structures

**Fig. 8:**
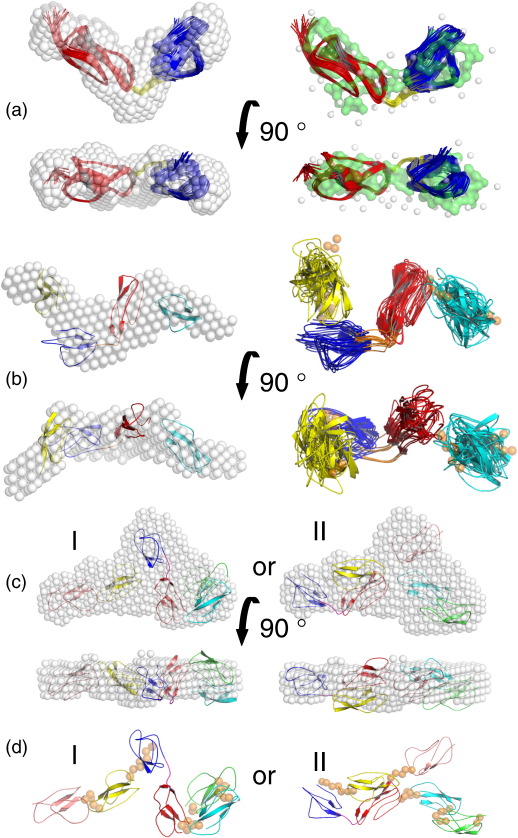
Modelling from SAXS data. (a) Overlay of DAMMIF-derived ab initio shape envelope (left) and GASBOR-derived dummy residue model (right) with the NMR-derived ensemble of fH12–13. (b) Overlay of the DAMMIF-derived ab initio shape envelope with the most typical BUNCH-derived model of fH11–14 (left); ensemble of 10 BUNCH models for fH11–14 (right). (c) Overlay of the DAMMIF-derived ab initio shape envelopes with the two most typical BUNCH models of fH10–15 (I and II). (d) The two most typical BUNCH models of fH10–15 (I and II). In (a)–(c), lower views are rotated 90° clockwise about the horizontal axis. The CCPs are shown in green (CCP 10), cyan (CCP 11), red (CCP 12), blue (CCP 13), yellow (CCP 14), and pink (CCP 15), with orange spheres representing linker residues modelled by BUNCH as a chain of dummy atoms.

Both ab initio shape reconstructions and SAXS-based rigid-body modelling were employed to independently determine the overall low-resolution structures of fH12–13, fH11–14, and fH10–15. In excellent agreement with NMR, the structures of fH12–13 determined by two ab initio modelling programs employing beads and dummy residues are essentially vee-shaped (Fig. 8a).

A more detailed modelling of the larger fH constructs was conducted using BUNCH^47^ to optimise a spatial distribution of structured domains and linkers (the latter represented as dummy residues) to best fit the SAXS data. The BUNCH-derived models generated for fH11–14 show a reproducible spatial distribution of the CCPs, with minor variation in their relative orientations (Fig. 8b). The long axes of the modules trace a zigzag path within a plane, compatible with the ab initio shape envelope. Modules 12 and 13 form closer contacts with flanking modules (CCPs 11 and 14) than with each other. The most typical model provides a very good fit to the experimental data with χ = 1.18 and, importantly, the fit does not improve when the CCP 12 and CCP 13 domains are treated as two separate rigid bodies. This observation further validates the use of the NMR-derived model of fH12–13 as a single rigid body in the analysis and suggests that the ∼ 80° tilt angle between CCPs 12 and 13 is conserved in the context of the larger construct.

The most typical fH11–14 BUNCH model was initially used as a single rigid body in the BUNCH analysis of fH10–15. Poor fits (2.13 < χ < 3.16) to the SAXS data suggest that the conformation of fH11–14 in solution differs from that of its constituent CCPs in the context of the fH10–15 construct. Thus, further rigid-body modelling was conducted using the NMR-derived structure of fH12–13. Whilst this strategy leads to generation of a large number of possible arrangements of CCPs, the resulting models of fH10–15 fall into two distinct clusters (Fig. 7c). These clusters share overall similarities, including the planar zigzag nature of the path described by the long axes of the modules, but they differ in the relative orientation of the central CCP 12–13 bimodule with respect to the flanking domains. In the first case (χ_RB_ = 1.35), CCPs 12 and 13 are oriented approximately perpendicular to the long axis of the fH10–15 molecule, producing a kink in the structure (Fig. 8d). In the second case (χ_RB_ = 1.30), the CCP 12–13 bimodule is oriented along the long axis, whilst a nearly − 180° tilt occurs between modules 13 and 14 (Fig. 8d). Note that BUNCH was also run by simultaneously fitting both fH10–15 and fH12–13 SAXS data without constraining CCP 12 and CCP 13 subunits according to the NMR-derived structure. A larger degree of variability between models was observed, however, and the fits were not improved (χ_RB_ = 1.29 and 1.99 for the fit to the most typical model using the fH10–15 and fH12–13 data, respectively).

## Discussion

The structural biology of the N-terminal and C-terminal regions of fH that encompas the known binding sites for its principal protein and carbohydrate ligands, has been studied extensively; the medium-resolution or high-resolution structures of all eight N-terminal CCPs have been experimentally determined {in fragments: fH1–2 [Protein Data Bank (PDB) ID 2RLP], fH2–3 (PDB ID 2RLQ), fH1–4 (PDB ID 2WII), and fH5 and fH6–8 (PDB ID 2UWN)} by NMR or crystallography, as have structures of four of the C-terminal six modules [fH15–16 (PDB ID 1HFH) and fH19–20 (PDB IDs 2BZM and 2G7I)]. The central region of fH, on the other hand, represents an unexplored structural territory.

We have now provided initial structural insights into the central CCPs of fH. The structure of module 12 is similar to those of other CCP structures. Indeed, experimentally derived and modelled (by homology)^42^ structures of fH CCP 12 are very similar overall (C^α^ RMSD = 1.4 Å). Module 13, the smallest CCP within the RCA family, has a highly divergent structure (Fig. 5); it thus joins a small group of structurally divergent modules (all of which diverge in different ways) within fH that includes CCPs 7 and 20.^42^ However, unlike these modules, CCP 13 (in the context of fH12–13, fH13–15, fH8–15, and fH10–15) has been shown not to bind to the model glycosaminoglycan heparin or to enzymatically generated fragments of heparan sulphate under physiological conditions.^27^ This is despite CCP 13 having one face that carries an extensive positive charge (whilst the other is predominantly neutral). It seems likely that such a divergent structure and striking electrostatic properties have evolved to serve a specialised but as yet unknown purpose. In total, we now know the structures of 14 CCPs in fH; however, our knowledge of how CCPs are spatially organised to form a functional complement regulator^48,49^ lags behind.

Before the current work, detailed structural knowledge was available for seven intermodular interfaces (fH1–2, fH2–3, fH3–4, fH6–7, fH7–8, fH15–16, and fH19–20) that feature intermodular linkers of between three and five residues in length. Leaving aside the possibility of overestimation of flexibility (e.g., in the structure of fH15–16 based on earlier NMR studies with no isotopic enrichment of the sample^50^) or overestimation of rigidity (e.g., in fH3–4, fH6–7, and fH7–8 derived from crystal structures^31,51^ without corroboration by NMR), the arrangement of neighbouring modules in all of these fragments may be classified as “end-to-end.”

Thus, none of the structures of fH fragments examined previously suggests large tilt angles (on average) between neighbouring CCPs; moreover, very similar arrangements of the three N-terminal modules of fH were observed both in solution^28^ and in complex with C3b,^51^ implying that modular organisation within the non-complexed fragments likely reflects the architecture of the functioning molecule, at least in some cases. Importantly, the intermodular linkers amongst this set of known CCP structures are between three and five residues in length; this may be compared to linker lengths of six, six, eight, seven, and five for the five linkers (in order) in human fH10–15 (i.e., of the 358 residues within fH10–15, nearly one-tenth lie in linkers). In this respect, it is noteworthy that the eight residues between the two CCPs at the N-terminus of complement receptor type 2 promoted a side-by-side arrangement of these modules^52^ (tilt angle = 142°; distance between the centres of mass of the two modules = 2.2 nm), as do the six linking residues between modules 2 and 3 of fB^53^ and complement C2.^54^ These observations had suggested the hypothesis that a similar side-by-side arrangement might be found between fH CCPs 12 and 13, which are joined by an eight-residue linker; however, long linkers also provide the theoretical potential for greater flexibility between modules, leading to an opposing hypothesis.

The current study falsifies both hypotheses, since fH modules 12 and 13 are neither side-by-side nor flexibly associated. Numerous intermodular and linker-to-module NOEs and the good convergence of the ensemble of solved structures, backbone relaxation data, and low-resolution data from size-exclusion chromatography, AUC, and SAXS all support the contention that the two modules exhibit a defined mutual tilt of ∼ 80°, burying a surface area of ∼ 560 Å^2^. Key interface residues (T724, I726, Val745, Ala746, Ile747, Leu750, His773, and Asn794) are conserved (or conservatively replaced) amongst orthologues (Fig. 1), so this arrangement of CCPs 12 and 13 of fH is likely conserved across species; on this basis, it is also highly likely to occur between CCPs 5 and 6 of fH-related protein 5 (Fig. 1). The even-and-odd mode (EOM) analysis of the SAXS data independently verifies the rigid nature of the long linker between CCP modules 12 and 13. It also indicates that the fH11–14 and fH10–15 substructures of fH are compact and rigid. Thus, the central CCPs of fH do not provide a flexible tether between the N-terminal region and the C-terminal region.

It was initially speculated that the CCP 12–CCP 13 tilt angle might be one of a succession of same-direction tilts between adjacent modules in the middle of fH. If this were the case, addition to fH12–13 of CCPs 11 and 14 (creating fH11–14) would produce a horseshoe-shaped entity or a spiral and would not result in a more extended structure. We put these predictions to test by comparing fH12–13 with fH11–14 using AUC and SAXS. Both techniques show that fH11–14 is more extended (*D*_max_ = 10.5 ± 0.5 nm) than fH12–13 (*D*_max_ = 7.0 ± 0.5 nm). Furthermore, the use of the NMR-derived fH12–13 structure in the rigid-body modelling of the SAXS data for fH11–14 produced a relatively well-converged set of conformers, allowing an approximation of intermodular tilts to be made. These are consistent with a compact conformation in which tilt angles between modules 11 and 12, and between modules 13 and 14, are larger than the 80° tilt between CCPs 12 and 13. On the other hand, the tilt between modules 11 and 13 is small, consistent with a zigzag arrangement rather than a horseshoe shape for fH11–14. A similar comparison was made between fH11–14 and fH10–15 (*D*_max_ = 10.4 nm). In this case, the additional modules did not result in a more extended structure (compared to fH11–14), implying that modules 10 and 15 are folded in towards modules 11–14.

Previous SAXS studies applied to full-length fH^23,39,55^ implied a *D*_max_ of ∼ 34–35 nm; fH consists of 20 CCPs, each of a typical length (3.5–3.8 nm). Whilst these results clearly rule out a highly extended end-to-end arrangement of CCPs in fH, they provide no information on the tilt angles between specific modules.^39^ Low-resolution data for fragments fH1–5, fH6–8, and fH16–20^56,57^ were not consistent with compacted structures (in agreement with high-resolution structural information, where available): for example, the estimated maximum dimensions for fH1–5 and fH16–20 were approximately 15 and 18 nm, respectively (note that the fH16–20 construct had a 26-residue C-terminal expression artefact) compared to a calculated value of 18–20 nm for a fully extended sequence of five CCPs^57^ and the value of 10.5 nm (measured in the current work) for the six-module construct fH10–15. This relatively extended nature of the fH N-terminal and C-terminal regions, combined with a *D*_max_ of only ∼ 34–35 nm for the intact fH, implies the presence of compact arrangements towards the centre of the fH molecule, consistent with our current findings.

Of the two families of fH10–15 conformations that fit well to the SAXS data, one features a hairpin bend between CCPs 13 and 14, which are joined by a seven-residue linker in human fH. Several strands of circumstantial evidence support such an unusual arrangement of modules in this particular case: (i) the aberrant structure of the C-terminal portion of module 13 (Fig. 5) and lack of rigidity in its D–E loop; (ii) a lack of conservation (of sequence and length) in the 13–14 linker residues amongst orthologues (Fig. 1; all other linkers in fH are well conserved) (extensive direct module–module contacts could supplant a structural role for specific linker side chains); (iii) such a folded-back structure might be stabilised by (or might stabilise) contacts between non-neighbouring (in the sequence) modules, thus explaining why addition of CCPs 10 and 15 could bring about a rearrangement of modules 10–14; (iv) a hairpin bend between CCPs 13 and 14 could place the two GAG-binding modules CCPs 7 and 20 in close proximity to each other, as has been proposed previously;^23,30,38,39^ and (v) this arrangement positions the electropositive face of CCP 13 on the outside of the 13–14 turn at a potentially exposed extremity of the folded-back molecule. The other favoured conformation does not feature the hairpin turn between CCPs 13 and 14 but, like the first model, portrays a compact zigzag arrangement of modules.

In summary, whilst more studies are needed to define a unique conformation of fH10–15, these data exclude the possibility that modules 10–15 act as a flexible tether between N-terminal and C-terminal binding sites. Rather, the central modules of fH form a rigid compact substructure in which the highly deviant CCP 13 is embedded. This module has an unusual distribution of charged groups at its surface, suggestive of a specific recognition site for an unknown ligand. Thus, a passive, purely structural role for the central portion of fH seems unlikely.

## Materials and Methods

### Protein production and validation

The expression of fH12–13 (fH residues 690–804), fH11–14 (residues 629–865), and fH10–15 (residues 568–927) in recombinant strains of *Pichia pastoris* was described previously.^27^ For the current work, isotopically enriched samples of fH12–13 were generated as follows. The fH12–13-overexpressing strain of *P. pastoris* was cultured in a 1.25-L vessel on a Bioflo 3000 fermentor (New Brunswick Scientific, Edison, NJ). For ^15^N labelling, cell mass was grown in 0.6 L of minimal medium containing 7.0 g of [^15^N]ammonium sulphate, 20 g of glucose, basal salts, trace elements, and vitamins. Prior to induction with methanol, 1.0 g of glycerol was added to facilitate derepression of the alcohol oxidase promoter.^58^ A ^13^C,^15^N-labelled sample was prepared following the same protocol as for ^15^N labelling, but [^13^C]glucose, glycerol, and methanol (Sigma-Aldrich, St. Louis, MO) were used.

Proteins were purified as described previously.^27^ In brief, cation-exchange or anion-exchange chromatography was followed by size-exclusion or heparin-affinity chromatography. N-linked glycans were removed by incubation with endoglycosidase H maltose-binding protein fusion protein (New England Biolabs, Ipswich, MA) either before purification or between the first purification step and the second purification step. Proteins were validated by peptide mass fingerprinting using matrix-assisted laser desorption ionisation time-of-flight mass spectrometry and (in the case of fH12–13), additionally, by mass determination on a Fourier transform ion cyclotron resonance mass spectrometer.

### NMR spectroscopy

A 620 μM sample of [^15^N,^13^C]fH12–13 in 20 mM potassium phosphate buffer (pH 6.6) plus 7% (vol/vol) D_2_O at 298 K was used for structure determination. A standard suite of NMR experiments enabled near-complete resonance assignments. Spectra were acquired on Bruker AVANCE 600-MHz and 800-MHz spectrometers equipped with 5-mm triple-resonance cryoprobes, using pulsed field gradients. Spectra were processed using TopSpin (Bruker version 1.3) or the Azara processing package [provided as part of the common computing protocol for NMR (CCPN) data model] and analysed using CCPN Analysis.^59^ Resonance assignments were accomplished using backbone [HNCACB,^60^ CBCA(CO)NH,^61^ HNCO,^62^ HN(CA)CO,^63^ HBHA(CBCACO)NH, and HBHANH^64^] and side-chain [H(CC)(CO)NH total correlated spectroscopy (TOCSY),^65^ (H)CC(CO)NH TOCSY,^63^ and H(C)CH TOCSY^66^] triple-resonance experiments, along with two-dimensional (HB)CB(CGCD)HD and (HB)CB(CGCDCE)HE aromatic side-chain experiments.^67^

### Assignment, structure calculations, and analysis

The excellent quality of fH12–13 NMR spectra is illustrated in Fig. 2. Spectral folding in the ^15^N dimension was employed to increase resolution. With the N-terminal cloning artefact (Ala-Gly) and one expression artefact unit (Glu-Ala), 89% of triple-resonance assignments, including 98% of backbone atoms, were completed. Of the theoretically assignable backbone atoms, the following are missing: Pro707 (CO), Val715 (CO), and Asn757 (CO, N, and HN).

Two out of five X-Pro linkage residues (Pro708 and Pro799) were defined as cis, whilst the remainder were defined as trans, as judged by chemical shift differences between Pro ^13^C^β^ and ^13^C^γ^ atoms;^68^ strong NOEs were detected between H^α^ (Xaa^*i*^^ − 1^) and H^α^ (Pro^*i*^), and between H^α^ (Xaa^*i*^^ − 1^) and HN (Xaa^*i *^^+ 1^), and no NOE was detected between H^α^ (Xaa^*i*^^ − 1^) and H^δ^ (Pro^*i*^). Based on accurate mass spectrometry, all eight cysteine residues were inferred to be in the oxidised state. Specific disulphide bridges were incorporated into the final rounds of structure calculations on the basis of NOEs (reinforced by a precedent established by previous studies of CCPs).

The ^15^N-edited NOE spectroscopy spectrum was assigned manually to near-completion, and ∼ 10% of peaks in a ^13^C-edited NOE spectroscopy spectrum were also assigned manually. The remaining NOEs were assigned within CYANA 2.1,^69^ which combines automated assignment and structure calculation. Upper limits of distance constraints generated from a seven-cycle routine within CYANA were transferred into the program Crystallography and NMR System,^70^ using the program Format Converter within the CCPN suite,^59^ in order to ultimately perform structure refinement using explicit water. Whilst no hydrogen-bond restraints were used in the structure calculation, a hydrogen/deuterium exchange experiment was performed to cross-validate 22 hydrogen bonds inferred from the final structure. Lyophilised protein was transferred to 99.9% (vol/vol) deuterated buffer, and slowly exchanging amide protons were identified in a ^15^N,^1^H HSQC spectrum collected 30 min after exposure to D_2_O. All of the slowly exchanging NH signals were engaged in hydrogen bonds, except for the Trp738 side-chain NH, which is buried in CCP 12. The quality of the data and structures was further analysed using WHAT IF^71^ and PROCHECK^72^ (see Table 1).

Secondary structure elements were identified using STRIDE.^41^ Surface potentials were determined using GRASP^43^ and the MOLCAD module^73^ of SYBYL v6.9 (Tripos Associates, St. Louis, MO). The solvent-accessible surface area (SA) and the buried surface area at the intermodular junctions were calculated using GETAREA.^20^ The buried surface area was computed as (SA module_12_ + SA module_13_) − SA bimodule_12–13_, where CCP 12 was considered to encompass one residue before its Cys^I^ and four residues after its Cys^IV^ (i.e., Thr690-Asp748); CCP 13 boundaries were considered the fourth residue before its Cys^I^ and one residue after its Cys^IV^ (i.e., Lys749-Ser804), and the bimodule consists of Thr690-Ser804. Intermodular angles for the ensemble of NMR-derived structures were determined as described previously,^18,50^ using (for a reference *x*-axis) a vector between the principal axis of the inertia tensor (the *z*-axis) and the C^α^ of the conserved Trp738 (CCP 12) or Trp797 (CCP 13), with module boundaries defined as Cys^I^ and Cys^IV^. Combinatorial extension^74^ was employed to compare each experimentally determined CCP structure within the complement system against the closest-to-mean individual structures of CCPs 12 and 13. Structural superimpositions were performed using MAMMOTH-mult^19^ then depicted using RasMol v2.7.3.^75^

### Relaxation measurements

Measurements of backbone ^15^N *T*_1_ and *T*_2_^76^ and ^1^H,^15^N NOEs^77^ were conducted at 600 MHz using 0.6 mM samples in 20 mM potassium phosphate buffer (pH 6.6). The delays used for measurements of *T*_1_ and *T*_2_ were 51.1, 51.1, 301.1, 501.1, 701.1, 801.1, 901.1, and 1001.1 ms, and 16.0, 16.0, 48.0, 80.1, 112.1, 128.1, 144.1, and 160.1 ms, respectively. For heteronuclear NOE measurement, a reference experiment was recorded with a 5-s relaxation delay, whilst a second spectrum was recorded with ^1^H saturation achieved by a train of ^1^H 120° pulses applied for the last 3 s of the 5-s delay. NMR data were processed using the AZARA suite of programs and assigned using Analysis. Where possible, a single-exponential decay was fitted to the extracted peak heights for each residue to obtain the relaxation rates using non-linear fitting. Peaks from the following residues were excluded due to overlap: Ser706, Tyr710, Tyr711, Cys744, Leu761, His773, Asn802, and Cys803; signals from the backbone amides of the following residues were too weak to allow measurement of relaxation parameters: His735, Gly736, Ile759, His764, Lys769, and Gly783.

### Size-exclusion chromatography

Samples of fH10–15 (82 μM), fH11–14 (118 μM), and fH12–13 (15 μM) [in 0.5 ml of potassium phosphate buffer (pH 6.6) supplemented with 500 mM NaCl] were loaded separately onto a Superdex 75 preparative-grade size-exclusion column (GE Healthcare, Piscataway, NJ), and elution profiles (monitored at 280 nm; theoretical extinction coefficients of 60,900, 47,400, and 28,500, respectively) were compared to the elution profiles of standards (bovine serum albumin, ovalbumin, chymotrypsinogen, ribonuclease A, and cyanocobalamin) applied under the same conditions.

### Analytical ultracentrifugation

Sedimentation velocity analyses were performed in a Beckman XL-A analytical ultracentrifuge at a rotor speed of 45,000 rpm, with a sample volume of 0.4 ml and a protein concentration of 0.2–0.4 mg ml^− 1^ in PBS (pH 7.5) at 293 ± 0.5 K. A series of radial scans with absorbance at 279 nm across the centrifuge cell was recorded and saved to disk via the Beckman-Coulter Proteome Lab software. The first scan was performed immediately upon attainment of the set rotor speed, and 80 subsequent scans were recorded at 2-min intervals thereafter. The total data set thus generated was analysed via the package SEDFIT^78^ to yield values of the *s*-distribution parameter *c*(*s*^⁎^) as a function of sedimentation coefficient (*s*). For non-linear fitting of the optical density values at 273 nm as a function of radial distance in SEDFIT, the resolution was set to 150, with a confidence factor set to 0.68. Over a series of fits, an average value for the frictional ratio (*F*, which relates to the “axial ratio” and pertains to the shape of the molecule) was determined; this was used as a default for all fittings to minimise artefactual variations. The baseline, meniscus, and cell base radial positions were floated during the fitting procedure. The final profile, stored as a continuous distribution, was analysed using the program proFIT (Quantum Soft, Zurich). The maximal value of the *c*(*s*) function was used to define the sedimentation coefficient of the species concerned. A value of the partial specific volume ($\overset{―}{v}$) (in ml g^− 1^) was computed via the program SEDNTERP†, which was also employed to compute the density and viscosity properties of the buffer solution, enabling the correction of raw *s* values to standard conditions of solvent viscosity and density (i.e., to *s*_20,w_ values).

The number of peaks in the fitted profile was used to infer the number of species present in the solution, with the area under each peak interpreted as reflecting the relative concentration of that species. The diffusion coefficient (an inverse function of the frictional ratio *F*) of each species was estimated via a Lamm equation fit in SEDFIT. From each value of *F* determined, it was straightforward to compute, on the basis of an assumed ‘typical’ (for protein) value of solvation (1.4, vol/vol), an estimate for the overall ‘shape’ of the solute particle, modeled as a prolate ellipsoid of revolution. SEDNTERP was employed to facilitate this computation.

### Small-angle X-ray scattering

Synchrotron radiation X-ray scattering data were collected at the X33 beamline of the European Molecular Biology Laboratory (DESY; Hamburg),^79,80^ using a MAR345 image plate detector (MarResearch, Norderstedt, Germany) and 120 s of exposure time. Solutions of fH constructs were measured at 10 °C in 50 mM potassium phosphate buffer (pH 7.4) at protein concentrations of 0.7, 1.8, and 3.5 mg ml^− 1^ (fH12–13), 0.7 and 1.6 mg ml^− 1^ (fH11–14), and 2.4, 5.1, and 10.5 mg ml^− 1^ (fH10–15). The sample-to-detector distance was 2.7 m, covering a range of momentum transfer 0.08 < *s* < 5.0 nm^− 1^ (*s* = 4πsinθ/λ, where 2θ is the scattering angle, and λ = 0.15 nm is the X-ray wavelength). Based on a repeat 120-s exposure, no detectable radiation damage occurred. Data from the image plate were normalised to the incident beam intensity and averaged, and the scattering of buffer solutions was subtracted. Difference curves were scaled for solute concentration. All data manipulations were performed using the PRIMUS software package.^81^

The forward scattering *I*(0) and the radius of gyration *R*_g_ were determined from Guinier analysis,^82^ assuming that, at very small angles (*s* < 1.3/*R*_g_), the intensity is represented as *I*(*s*) = *I*(0)exp(−(s*R*_g_)^2^/3). These parameters were also estimated from the full scattering curves using the indirect Fourier transform method implemented in the program GNOM,^83^ along with the distance–distribution function *p*(*r*) and the maximum particle dimensions *D*_max_. Molecular masses of solutes were estimated from SAXS data by comparing extrapolated forward scattering with that of a reference solution of bovine serum albumin.

Due to the uncertainty in molecular mass estimation from SAXS data that results from uncertainty in the measured protein concentrations, an excluded volume of the solutes was determined from the ab initio modelling program DAMMIF.^84^ This estimation is independent of protein concentration and can be obtained in an automated fashion with minimal user bias. For globular proteins, this hydrated particle volume (in nm^3^) is approximately 1.5–2 times the molecular mass (in kDa).

### Analysis of the NMR ensemble of fH12–13

The fit of the NMR ensemble of fH12–13 to the SAXS data was conducted using the program CRYSOL.^85^ CRYSOL calculates the partial scattering amplitudes of proteins from their atomic coordinates, taking into account the hydration layer and the excluded solvent volume.

### Analysis of interdomain flexibility by the ensemble optimisation method

Analysis of interdomain flexibility and the size distribution of possible conformers, consistent with the measured scattering data for fH12–13, fH10–14, and fH10–15, was conducted using the ensemble optimisation method.^45^ This method selects an ensemble of possible conformations from a pool of randomly generated models, using CRYSOL (to calculate the theoretical scattering profiles) and a genetic algorithm GAJOE (to select the representative set). The input structures for an analysis of the scattering data employing ensemble optimisation used the following as rigid bodies: the NMR-derived structure of CCP 15 (PDB ID 1HFI) and fH12–13 (this study), and homology models of CCPs 10, 11, and 14 using the program Modeler 9v1. Linkers between the modules were represented as a flexible chain of dummy residues.

### Ab initio shape determination and molecular modelling

Low-resolution shape envelopes for the fH constructs were determined using the ab initio bead modelling program DAMMIF.^84^ DAMMIF represents the particle as a collection of *M* (≫ 1) densely packed beads inside an adaptable and loosely constrained search volume compatible with the experimentally determined *R*_g_. In contrast to the bounded search volume used in the bead modelling program DAMMIN,^86^ an unrestricted search volume that can grow or reduce in size during the simulated annealing procedure avoids artefactual boundary effects that may occur in the case of anisotropic particles. Each bead is randomly assigned to solvent (index = 0) or solute (index = 1), and the particle structure in solution is described by a binary string of length *M*. Disconnected strings of beads are rejected, and scattering amplitudes are calculated. Simulated annealing is then used to search for a compact model that minimises the discrepancy:

$$x^{2}=\sum_{k}\frac{1}{N-1}\sum_{j}\left[\frac{I_{\exp}(S_{j})-cI_{\text{calc}}(S_{j})}{\sigma (S_{j})}\right]$$

where *N* is the number of experimental points; *I*_exp_(*S_j_*) and *I*_calc_(*S_j_*) are the experimental and calculated intensities, respectively; *c* is a scaling factor; and σ(*S_j_*) is the experimental error at the momentum transfer *S_j_*. Low-resolution models were also determined from the ab initio dummy residue modelling program GASBOR,^87^ which represents the particle in solution as a protein-like chain of dummy residues, thus representing more accurately the internal structure of the particle than do the shapes determined from DAMMIF.

The results of multiple DAMMIF and GASBOR reconstructions were compared using the alignment program SUPCOMB^88^ to determine the most representative (typical) model from each of the ab initio methods. Averaged DAMMIF and GASBOR models were also determined using the program DAMAVER,^89^ and these models were adjusted such that they agree with the experimentally determined excluded volume using the program DAMFILT.^89^

Molecular modelling of fH constructs was conducted using, as rigid bodies and where appropriate, the same structures as those used in the EOM. Rigid-body models were generated for the fH12–13, fH11–14, and fH10–15 constructs using the program BUNCH,^47^ where linkers/loops between the individual CCP subunits are represented as flexible chains composed of dummy residues. Domain boundaries were defined as the first and the last cysteine residues in each CCP module. For BUNCH rigid-body modelling, either single scattering curves or multiple curves were used for refinement of the fH11–14 and fH10–15 constructs (i.e., the scattering curves of smaller fH constructs were used for simultaneous refinement). During the modelling procedure, no NOE between CCP modules was enforced. The results of multiple BUNCH runs were analysed using the programs SUPCOMB and DAMAVER to identify the most representative/typical models.

The sedimentation coefficient *s*_20__xw_ of the SAXS-generated bead models of all factor H constructs was computed by HYDROPRO.^44^ The values obtained for individual models and for the averaged models provided by DAMAVER were analysed to assess uncertainty in the SAXS-derived values of *s*_20xw_.

### Accession number

Structural data have been deposited in the PDB with accession code 2KMS (BioMagResBank entry number 16439).

## Figures and Tables

**Table 1:** Experimental input and statistics for the lowest-energy fH12–13 structures

Number of structures in ensemble^a^	20
Numbers of upper-limit distance constraints	
Intraresidue, |*i* − *j*| < = 1	1371
Medium range, 1 < |*i* − *j*| < 5	359
Long range, |*i* − *j*| > = 5	1181
Total	2911
Intermodular	10
Module 12 to linker	75
Module 13 to linker	75
	
Number of hydrogen bonds^b^	22
RMSD^c^	
Bimodule (12 + 13) (Å)	
All heavy atoms	1.64
Backbone heavy atoms	1.23
Module 12 (Å)	
All heavy atoms	0.88
Backbone heavy atoms	0.33
Module 13 (Å)	
All heavy atoms	1.10
Backbone heavy atoms	0.39
Intermodular angles {minimum–maximum [mean (S.D.)]}	
Skew (°)	78–91 [85 (4)]
Twist (°)	64–122 [91 (18)]
Tilt (°)	61–91 [78 (9)]
Ramachandran assessment	
Most favoured (%)	72.4
Additionally allowed (%)	22.2
Generously allowed (%)	2.9
Disallowed (%)	2.4
Coarse packing WHAT IF score^d^	− 1.671
	
Surface area buried between modules^e^ (Å^2^)	561^e^

a PDB ID 2KMS and BioMagResBank entry number 16439.

b Not used as input for structure calculation.

c Residues from Cys^I^ to Cys^IV^ of each module.

d Closest-to-mean structure.

e Calculated by breaking fH12–13 at the midpoint of the linker.

**Table 2:** Summary of SAXS and AUC data

Sample (molecular weight kDa^a^)	SAXS^b^							AUC^c^							
	*R*_g_ (nm)	*D*_max_ (nm)	*V*_p_ (nm^3^)	Molecular weight (kDa)	*s*_20xw_ (S)	χ_s_	χ_RB_	*s*_20,w_ (S)	Molecular weight (kDa)	$\overset{―}{v}$ (ml g^− 1^)	% mono	*F*_c(M)_	*F*_s, M_	*f*/*f*_min_	Axial ratio
fH12–13 (13.6)	2.2 ± 0.1	7.1 ± 0.5	20 ± 2.5	19 ± 2	1.6 ± 0.1	1.58	1.24	1.74 ± 0.03	16.8 ± 0.8	0.724	80 ± 1	1.33	1.20	1.00	1.00
fH11–14 (27.3)	3.1 ± 0.1	10.5 ± 0.5	38 ± 7	35 ± 4	2.5 ± 0.2	1.43	1.18	2.25 ± 0.04	26.5 ± 1.3	0.725	99 ± 1	1.42	1.48	1.23	4.75
fH10–15 (41.1)	3.1 ± 0.1	10.4 ± 0.5	68 ± 13	46 ± 6	3.4 ± 0.2	0.81	1.30	3.19 ± 0.05	40.5 ± 2.0	0.725	97 ± 1	1.35	1.37	1.14	3.39

a Molecular weights calculated from sequence.

b *R*_g_, *D*_max_, and *V*_p_ are the experimentally (SAXS) determined radius of gyration, maximum particle dimension, and hydrated particle volume, respectively. *s*_20xw_ is the sedimentation coefficient computed from the SAXS bead models. χ_s_ and χ_RB_ are the discrepancies between the experimental data and the computed scattering curves from the most typical ab initio and rigid-body models, respectively. The χ_RB_ shown for fH12–13 is the value of the best-fit NMR model to the SAXS data calculated using CRYSOL.

c *s*_20,w_, $\overset{―}{v}$, and % mono are the experimentally (AUC) determined sedimentation coefficient, partial specific volume, and percent monomer in the sample, respectively. *F*_c__(M)_ is the frictional ratio [calculated from the initial *c*(*s*^⁎^) fit]. *F*_s,M_ is the frictional ratio calculated from the sedimentation coefficient and the known (from sequence) molecular mass. *f*/*f*_min_ is the component of the frictional ratio that can be attributed to shape, on the basis that for a non-extended ‘globular’ protein, a value *F* = 1.20 (default value in SEDFIT) is generally applicable (for the computation of *f*/*f*_min_, the second and more reliable of the two estimates for *f*/*f*_0_ has been divided by 1.20).

## Appendix A Supplementary data

Fig. S1 Lipophilic surface representation of fH12–13. These view corresponds to those of Fig. 6a; the most hydrophilic and lipophilic surface features are labelled according to residue. There are no extensive hydrophobic patches on the surface of either module, but CCP 12 is characterised by a scattering of exposed lipophilic side chains. Fig. S2. Representative data from sedimentation velocity AUC. The sample shown is fH10–15. The upper frame shows a series of scans across the centrifuge cell; the first scan is shown in black, and subsequent scans are shown in rainbow colours. The lowest frame shows the *c*(*s*⁎) distribution of sedimentation rates (coefficients) for the population of protein molecules in the cell, as extracted from the scans in the upper frame using the fitting routine SEDFIT. The quality of the fit is evidenced (intermediate frames) by plots of the bitmap of the residuals above and the simple radial distribution of the residuals below. The area under the peak is proportional to the amount of protein, and the plot may be compared to an inverse size-exclusion chromatogram. The presence of a single (neo-Gaussian) peak is good evidence for the existence of a single species in this sample of fH10–15. Similar plots were obtained over the range of samples analysed. Calculated molecular masses and frictional ratios (relating to the “axial ratio” of the molecule) are shown in Table 2. Fig. S3. Factor H-orthologue alignment. Multiple sequence alignment of fH (all 20 CCPs) from a range of mammals. Orthologues were identified via a BLAST search (Altschul, 1997) against the UniProt database (Bairoch, 2005) using the NPS@ server (http://npsa-pbil.ibcp.fr/cgi-bin/npsa_automat.pl?page=/NPSA/npsa_server.html) (Combet, 2000). Their mature sequences were subsequently aligned using ClustalX (Thompson,1997), subjected to minor manual editing and displayed using BOXSHADE v3.21 (http://www.ch.embnet.org/software/BOX_form.html) (black background, strictly conserved; gray background, conservatively substituted; white background, non-conserved).The modules are individually labelled, and the locations of intermodular linker amino acid residues are highlighted with red boxes. The differences in linker lengths and the lack of conservation are shown. The CCP 13–CCP 14 linker is apparent in this representation.

## Footnotes

† http://www.rasmb.bbri.org
