# Supplementary material for: The Central Portion of Factor H (Modules 10–15) Is Compact and Contains a Structurally Deviant CCP Module
Source: J Mol Biol. 2010 Jan 8;395(1):105–22. doi: 10.1016/j.jmb.2009.10.010 (PMC2806952; doi:10.1016/j.jmb.2009.10.010)
Supplement: Fig. S1 — Lipophilic surface representation of fH12–13. These view corresponds to those of Fig. 6a; the most hydrophilic and lipophilic surface features are labelled according to residue. There are no extensive hydrophobic patches on the surface of either module, but CCP 12 is characterised by a scattering of exposed lipophilic side chains. Fig. S2. Representative data from sedimentation velocity AUC. The sample shown is fH10–15. The upper frame shows a series of scans across the centrifuge cell; the first scan is shown in black, and subsequent scans are shown in rainbow colours. The lowest frame shows the c(s⁎) distribution of sedimentation rates (coefficients) for the population of protein molecules in the cell, as extracted from the scans in the upper frame using the fitting routine SEDFIT. The quality of the fit is evidenced (intermediate frames) by plots of the bitmap of the residuals above and the simple radial distribution of the residuals below. The area under the peak is proportional to the amount of protein, and the plot may be compared to an inverse size-exclusion chromatogram. The presence of a single (neo-Gaussian) peak is good evidence for the existence of a single species in this sample of fH10–15. Similar plots were obtained over the range of samples analysed. Calculated molecular masses and frictional ratios (relating to the “axial ratio” of the molecule) are shown in Table 2. Fig. S3. Factor H-orthologue alignment. Multiple sequence alignment of fH (all 20 CCPs) from a range of mammals. Orthologues were identified via a BLAST search (Altschul, 1997) against the UniProt database (Bairoch, 2005) using the NPS@ server (http://npsa-pbil.ibcp.fr/cgi-bin/npsa_automat.pl?page=/NPSA/npsa_server.html) (Combet, 2000). Their mature sequences were subsequently aligned using ClustalX (Thompson,1997), subjected to minor manual editing and displayed using BOXSHADE v3.21 (http://www.ch.embnet.org/software/BOX_form.html) (black background, strictly conserved; gray background [file mmc1.pdf]

SUPPLEMENTARY DATA

Figure S1. *Lipophilic surface representation of fH12-13*. These views corresponds to those of Figure 6A; the most hydrophilic and lipophilic surface features are labelled according to residue. There are no extensive hydrophobic patches on the surface of either module, but CCP 12 is characterised by a scattering of exposed lipophilic side-chains.

Figure S2. *Representative data from sedimentation velocity AUC*. The sample shown is fH10-15. The upper frame shows a series of scans across the centrifuge cell - the first scan is shown in black and subsequent scans in rainbow colours. The lowest frame shows the c(s*) distribution of sedimentation rates (coefficients) for the population of protein molecules in the cell, as extracted from the scans in the upper frame using the fitting-routine SEDFIT. The quality of the fit is evidenced (intermediate frames) by plots of the bitmap of the residuals above and the simple radial distribution of the residuals below. The area under the peak is proportional to the amount of protein, and the plot may be compared to an inverse size-exclusion chromatogram. The presence of a single (neo-Gaussian) peak is good evidence for the existence of a single species in this sample of fH10-15. Similar plots were obtained over the range of samples analysed. Calculated molecular weights and frictional ratios (relates to the "axial ratio" of the molecule) are shown in *Table 2*.

Figure S3. *Factor H-orthologue alignment***.** Multiple-sequence alignment of fH (all 20 CCPs) from a range of mammals. Orthologues were identified via a BLAST search (Altschul, 1997) against the UniProt database (Bairoch, 2005) using the NPS@ server (http://npsa-pbil.ibcp.fr/cgi-bin/npsa_automat.pl?page=/NPSA/npsa_server.html) (Combet, 2000). Their mature sequences were subsequently aligned using ClustalX (Thompson, 1997), subjected to minor manual editing and displayed using BOXSHADE v3.21

(http://www.ch.embnet.org/software/BOX_form.html) (black background = strictly conserved; grey background = conservatively substituted; white background = non-conserved). The modules are individually labelled and the locations of inter-modular linker amino acid residues are highlighted with red boxes. The differences in linker-lengths, and the lack of conservation, the CCP 13-CCP 14 linker is apparent in this representation.

References for supplementary data:

Altschul, S.F., Madden, T.L., Schaffer, A.A., Zhang, J., Zhang, Z., Miller, W., and Lipman, D.J. 1997. Gapped BLAST and PSI-BLAST: a new generation of protein database search programs. *Nucleic Acids Res* **25:** 3389-3402.

Berman HM, Westbrook J, Feng Z, Gilliland G, Bhat TN, Weissig H, Shindyalov IN, Bourne PE 2000. The protein databank *Nucleic Acids Res* **28**:235-242.

Bairoch, A., Apweiler, R., Wu, C.H., Barker, W.C., Boeckmann, B., Ferro, S., Gasteiger, E., Huang, H., Lopez, R., Magrane, M., et al. 2005. The Universal Protein Resource (UniProt). *Nucleic Acids Res* **33:** D154-159.

Combet, C., Blanchet, C., Geourjon, C., and Deleage, G. 2000. NPS@: network protein sequence analysis. *Trends Biochem Sci* **25:** 147-150.

Shindyalov, I.N., Bourne, P.E. 1998. Protein structural alignment by incremental combinatorial extension of the optimal path *Protein Eng* **11**:739-747

Thompson, J.D., Gibson, T.J., Plewniak, F., Jeanmougin, F., and Higgins, D.G. 1997. The CLUSTAL_X windows interface: flexible strategies for multiple sequence alignment aided by quality analysis tools. *Nucleic Acids Res* **25:** 4876-4882.

Table S1: **Table 1: Pair-wise CCP module structural comparisons of FH~12 and FH~13.**

**Protein~module number (PDB code (Berman et al., 2000))**	**FH~12 RMSD in Å (alignment length, gaps included)**	**FH~13 RMSD in Å (alignment length, gaps included)**
FH~12		3.54 (46)
FH~13	3.54 (46)	
C1r~01 (1GPZ)	2.11 (58)	3.07 (53)
C1r~02 (1GPZ)	1.95 (38)	3.95 (38)
C1s~02 (1ELV)	1.71 (48)	4.13 (51)
C2~01 (3ERB)	2.64 (49)	4.13 (55)
C2~02 (3ERB)	2.62 (58)	3.18 (57)
C2~03 (3ERB)	1.99 (56)	3.71 (49)
C4BPα~01 (2A55)	2.27 (58)	3.43 (57)
C4BPα~02 (2A55)	2.11 (57)	4.73 (58)
CR1~15 (1GKN)	2.32 (57)	3.03 (46)
CR1~16 (1GKN)	2.17 (57)	3.68 (52)
CR1~17 (1GKG)	1.88 (57)	3.48 (67)
CR2~01 (1LY2)	1.66 (58)	4.13 (58)
CR2~02 (1LY2)	1.41 (57)	3.29 (48)
DAF~01 (1OK3)	2.32 (58)	3.31 (58)
DAF~02 (1OK3)	1.83 (58)	2.73 (50)
DAF~03 (1H03)	2.26 (57)	3.78 (52)
DAF~04 (1H03)	1.59 (57)	3.26 (51)
FB~01 (2OK5)	3.26 (50)	4.86 (51)
FB~02 (2OK5)	2.34 (58)	4.16 (56)
FB~03 (2OK5)	2.10 (56)	3.58 (50)
FH~01 (2RLP)	2.23 (56)	3.05 (47)
FH~02 (2RLQ)	2.11 (55)	4.17 (55)
FH~03 (2RLQ)	1.98 (56)	3.86 (47)
FH~04 (2WII)	1.82 (57)	2.79 (56)
FH~05 (not deposited)	2.41 (53)	3.16 (45)
FH~06 (2UWN)	2.69 (57)	2.79 (50)
FH~07 (2UWN)	3.16 (57)	3.55 (52)
FH~08 (2UWN)	1.91 (51)	3.41 (59)
FH~15 (1HFH)	1.93 (57)	2.89 (49)
FH~16 (1HFH)	1.66 (56)	4.10 (54)
FH~19 (2G7I)	1.40 (58)	3.74 (55)
FH~20 (2G7I)	2.12 (55)	3.00 (48)
MASP1~01 (3GOV)	2.29 (58)	2.85 (63)
MASP1~02 (3GOV)	2.00 (58)	4.26 (61)
MASP2~01 (1ZJK)	2.10 (58)	3.26 (54)
MASP2~02 (1ZJK)	1.81 (58)	4.16 (59)
MCP~01 (1CKL)	2.36 (57)	3.78 (46)
MCP~02 (1CKL)	2.53 (52)	3.85 (63)
VCP~01 (1G40)	2.60 (58)	3.84 (60)
VCP~02 (1G40)	2.31 (51)	3.34 (52)
VCP~03 (1G40)	2.11 (57)	3.05 (59)
VCP~04 (1G40)	2.12 (55)	3.25 (57)

[1]Comparison of individual closest-to-mean structures of FH~12 and FH~13 versus all other individual CCPs of known structure within the complement system based upon C_α RMSD values using structural alignment program CE (Shindyalov and Bourne, 1998). For each

CCP, inclusive module boundaries were one residue before Cys^{I} and the third residue after Cys^{III}. In cases where structures have been solved by both NMR and X-ray diffraction, the higher resolution X-ray structure was used for comparison. Where both liganded and unliganded structures were available, the highest resolution unliganded X-ray or NMR structure was used. A few residues were missing in the crystal structure of C1r~02, and hence in this case, the structure with the most determined residues was employed for both modules. Colour key used in table: Blue: 0 - 1.99 Å; Green: 2.00 – 2.99 Å; Red: 3.00 – 3.99 Å; Black: >= 4.00 Å; Brown: Alignment lengths < 40 amino acids. Abbreviations used in Table: $C4BP_{\alpha}$ = C4b-binding protein α-chain; CR = complement receptor; DAF = decay-accelerating factor; FB = factor B; FH = factor H; MASP1,2 = mannan-binding lectin-associated serine proteases 1 and 2; MCP = membrane cofactor protein; VCP = Vaccinia virus complement control protein. Some residues were not present (solved) in the electron density map for the C1r~02 module crystal structure, and this explains the short structural alignment length (shown in brown).

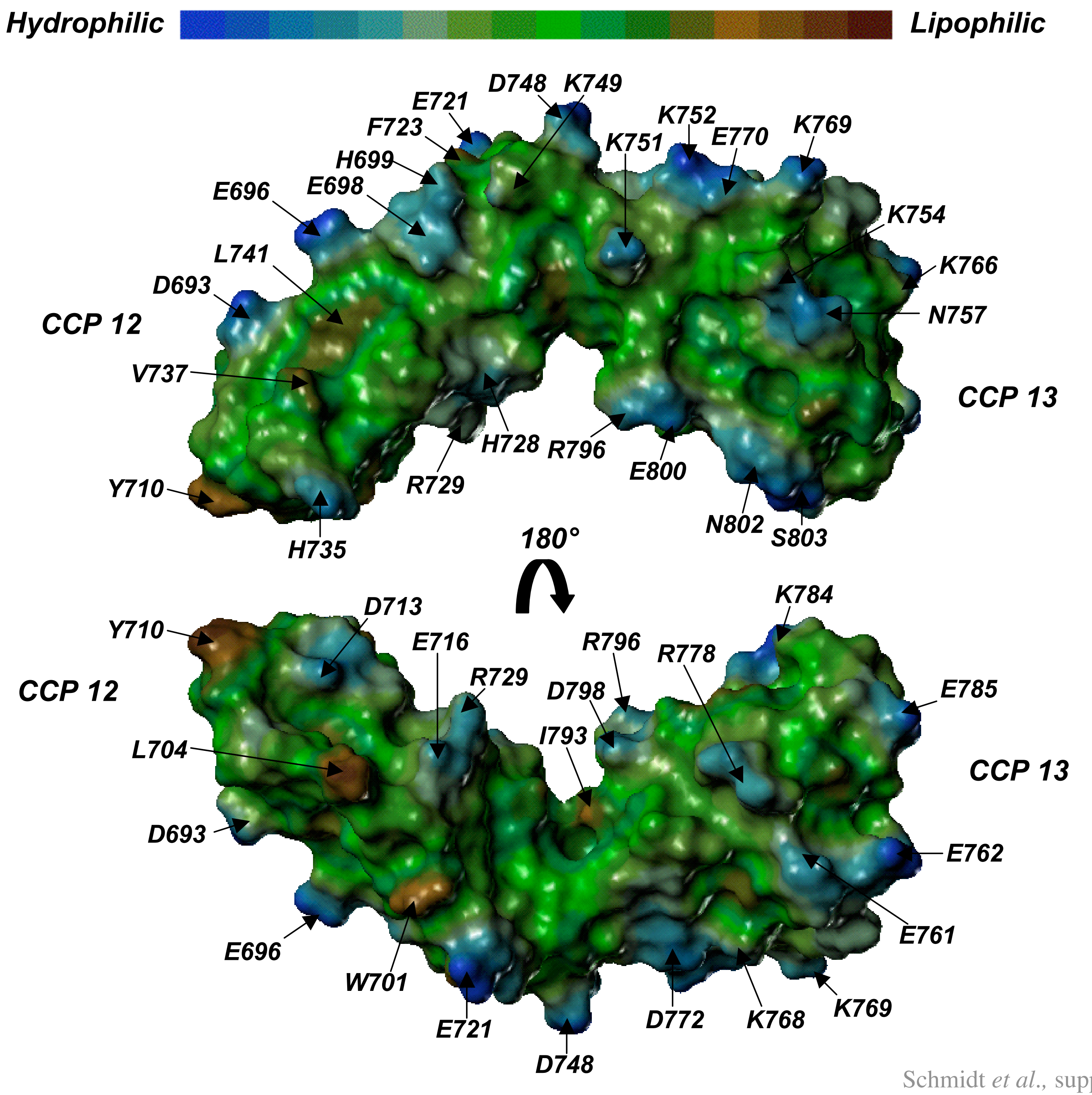

Schmidt *et al.*, supplementary data Figure 1

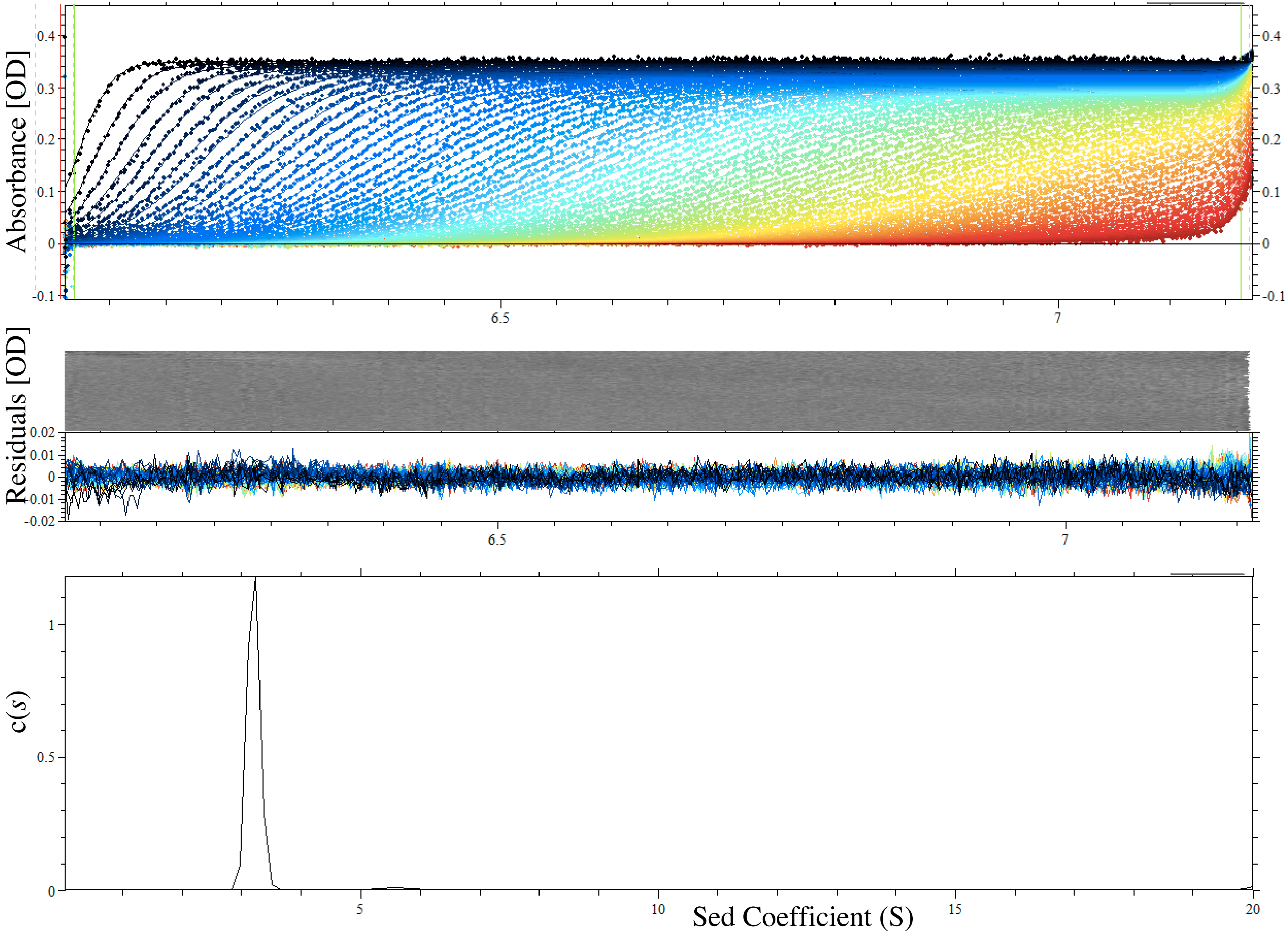
Absorbance [OD]
0.4
0.3
0.2
0.1
0
-0.1
6.5
7
Residuals [OD]
0.02
0.01
0
-0.01
-0.02
6.5
7
c(s)
1
0.5
0
5
10
15
20
Sed Coefficient (S)

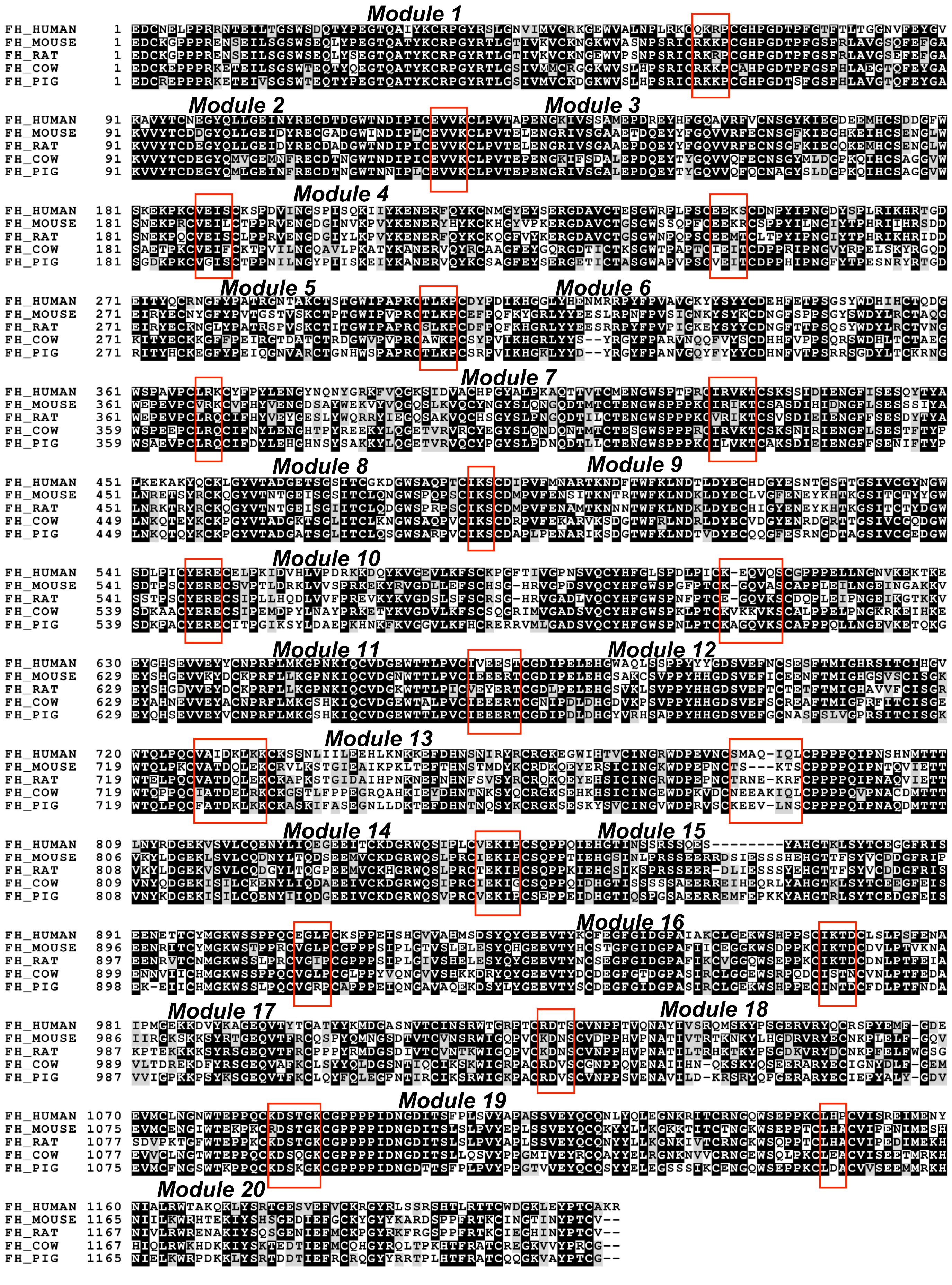
Module 1
Module 2
Module 3
Module 4
Module 5
Module 6
Module 7
Module 8
Module 9
Module 10
Module 11
Module 12
Module 13
Module 14
Module 15
Module 16
Module 17
Module 18
Module 19
Module 20
FH_HUMAN
FH_MOUSE
FH_RAT
FH_COW
FH_PIG
